# Association of the geriatric nutritional risk index with poor outcomes in patients with coronary revascularization: a cohort study

**DOI:** 10.3389/fcvm.2024.1442957

**Published:** 2024-12-24

**Authors:** Beili Xie, Yue Shi, Mingwang Liu, Zhidie Jin, Wei Wen, Yuxin Yan, Mengjie Gao, Lulian Jiang, Lin Yang, Jiangang Liu, Dazhuo Shi, Fuhai Zhao

**Affiliations:** ^1^ Cardiovascular Department, Xiyuan Hospital, China Academy of Chinese Medical Sciences, Beijing, China; ^2^ Graduate School of Beijing University of Chinese Medicine, Xiyuan Hospital, Beijing, China; ^3^ National Clinical Research Center for Chinese Medicine Cardiology, Xiyuan Hospital, Beijing, China

**Keywords:** nutritional risk index in the elderly, coronary revascularization, mortality, acute kidney injury, MIMIC-IV database, cohort study

## Abstract

**Background:**

Poor nutritional status may affect outcomes after coronary revascularization, but the association between nutritional status and outcomes in patients undergoing coronary revascularization has not been fully evaluated. This study was based on the MIMIC-IV database to analyze the impact of baseline nutritional status on poor outcomes in patients with coronary revascularization.

**Methods:**

Patients with coronary revascularization were screened from the MIMIC-IV database. A geriatric nutritional risk index (GNRI) was calculated and used to divide patients into 4 groups: no malnutrition (*Q*4: ≥96.79), mild malnutrition (*Q*3: 90.85–96.78), moderate malnutrition (*Q*2: 86.37–90.84), and severe malnutrition (*Q*1: 86.37). The primary outcome measure was 28-day mortality, and the secondary outcome measures were AKI and length of hospital stay. Cox proportional hazards model, Kaplan-Meier survival analysis, restricted cubic spline (RCS), and multiple linear regression model were used for statistical analysis, respectively, to ensure the robustness of study results.

**Results:**

A total of 1,168 patients with coronary revascularization were included. The GNRI demonstrated a significant association with 28-day mortality in patients undergoing coronary revascularization. As a continuous variable, the GNRI exhibited a notable inverse correlation with mortality across unadjusted, partially adjusted, and fully adjusted Cox regression models [hazard ratios (HRs): 0.93, 0.94, 0.96, respectively; all *P* < 0.001]. When considered as a categorical variable, a low GNRI (first quartile, *Q*1) was significantly associated with elevated mortality risks (HRs: 2.64, 2.30, 1.82 in the unadjusted, partially adjusted, and fully adjusted models, respectively; all *P* < 0.05). Subgroup analysis revealed a more pronounced association in patients under 65 years of age (*P* for interaction = 0.014). Furthermore, reduced GNRI levels were also associated with an increased incidence of AKI and extended hospital lengths of stay.

**Conclusion:**

GNRI is associated with prognosis in patients with coronary revascularization. Patients with lower GNRI had higher 28-day mortality, greater risk of AKI, and longer hospital stays.

## 1 Introduction

Coronary revascularization, including percutaneous coronary intervention, coronary artery bypass grafting, and the combination of these two approaches (1), has brought about a revolutionary change in the treatment of coronary heart disease. However, the long-term outcomes of these therapeutic methods are not entirely favorable.

Research has demonstrated a considerable incidence of all-cause mortality and major adverse cardiovascular events (MACE) following coronary revascularization. Notably, over one-quarter of patients undergoing percutaneous coronary intervention (PCI) experience MACE, encompassing events such as death, non-fatal myocardial infarction (MI), or target vessel revascularization (TVR) (2). Head et al. (3) included 11 randomized trials to examine mortality between revascularization strategies and pooled data from 11,518 patients assigned to undergo PCI (*n* = 5,753) or CABG (*n* = 5,765). A meta-analysis revealed that the 5-year all-cause mortality rates were 11.2% following PCI and 9.2% after CABG. Consequently, it is imperative to implement preventive strategies aimed at identifying high-risk factors for adverse outcomes in patients undergoing coronary revascularization, with the ultimate goal of reducing mortality in this patient population.

Numerous factors influence the recovery and prognosis of patients following coronary revascularization, with malnutrition being a particularly significant concern. Malnutrition refers to a state of insufficient nutrient intake, absorption, or excessive depletion, or the consumption of a diet lacking in balance, leading to nutrient deficiencies. Recent evidence suggests that malnutrition is an important predictor of poor prognosis after cardiac surgery (4), and that pre-and post-operative nutritional supplementation can have a positive impact on myocardial glucose metabolism (5). Currently, a variety of scoring systems are used to assess the nutritional status of patients, with the GNRI (Global Nutritional Risk Assessment Index) being preferred for its accuracy and convenience (6). While studies have investigated the predictive utility of the GNRI for outcomes following PCI (7), research encompassing both PCI and CABG has yet to be reported.

MIMIC-IV database provides a large number of real clinical data of critically ill patients (8). An extensive body of research has meticulously explored databases, yielding numerous achievements with significant clinical application value. The objective of the present study was to examine the association between baseline nutritional status and clinical outcomes in patients undergoing coronary revascularization, utilizing the MIMIC-IV database. We posited that the GNRI could serve as a prognostic indicator for patients undergoing coronary revascularization, with the hypothesis that lower GNRI values would be correlated with poorer clinical outcomes.

## 2 Materials and methods

### 2.1 Data sources

Data were extracted from the Medical Information Mart for Intensive Care IV (MIMIC-IV) database (https://mimic.mit.edu). This database is open source, records Beth Israel Deaconess Medical Center ICU adult case data from 2008 to 2019, and de-identifies all patient private information, thus exempting ethical approval and informed consent requirements. Author Shi Yue completed an online course at the National Institutes of Health and passed the Human Research Participant Protection exam to gain access to the MIMIC-IV database.

### 2.2 Study population

Adult patients admitted to ICU for the first time undergoing coronary revascularization surgery, including PCI and CABG, were included. AKI was defined as an increase in Scr levels ≥0.3 mg/dl within 48 h or an increase in Scr ≥1.5-fold from baseline within 7 days, passing the Kidney Disease: Improving Global Outcomes (KDIGO) guideline criteria. Patients who lacked serum albumin, height, and weight within 24 h of admission to ICU were excluded.

### 2.3 Data acquisition

Demographic information, Sequential Organ Failure Assessment (SOFA) scores, laboratory parameters, comorbidities, interventions, outcome events, etc. were extracted using Structured Query Language (SQL) running PostgreSQL (version 13.7.2). Demographic information includes age, gender, and BMI. Laboratory parameters were selected within 24 h after first admission to ICU, including red blood cell count, white blood cell count, hemoglobin, platelets, serum creatinine (Scr), urea nitrogen (BUN), albumin, blood glucose, potassium, and blood sodium. Concomitant diseases include heart failure, chronic kidney disease (CKD), hypertension, diabetes mellitus (DM), sepsis, and malignancy. Interventions included renal replacement therapy (RRT), diuretics, and vasoactive drugs, and outcome events included 28-day mortality, AKI incidence, and length of hospital stay.

### 2.4 Outliers and missing value management

Variables with outliers are processed by the winsorize method using the STATA winsor2 command, with cut-off points of 1% and 99%. Missing values were imputed using multiple imputation methods. Variables with missing values of more than 25% were excluded, such as lipids, transaminases, etc.

### 2.5 Study groups and endpoints

GNRI is calculated as: 1.489× serum albumin (g/L) + 41.7 × body weight (kg)/ideal body weight (kg). Ideal weight was estimated according to Lorenz formula, i.e., ideal weight for men = height (cm) − 100 − [(height (cm) − 150)/4]; ideal weight for women = height (cm) − 100 − [(height (cm) − 150)/2.5]. When the actual weight/ideal weight ratio is greater than 1, set it to 1. All patients were divided into four groups according to GNRI quartiles, namely *Q*1 (86.37), *Q*2 (86.37–90.84), *Q*3 (90.85–96.78), and *Q*4 (≥96.79).*Q*4, *Q*3, *Q*2 and *Q*1 represent no malnutrition risk, mild, moderate and severe malnutrition risk, respectively.

The primary endpoint was mortality at 28 days, and secondary endpoints were the incidence of AKI and length of stay.

### 2.6 Statistical analysis

Continuous variables for this study are expressed as medians (interquartile ranges) and comparisons are made by Kruskal-Wallis H due to their non-normal distribution. Categorical variables were expressed as *n* (%) and comparisons were made using chi-square or Fisher's exact test.

Hazard ratios (HRs) and 95% confidence intervals (CIs) for GNRI and 28-day mortality, AKI were calculated using Cox proportional hazards models and adjusted for multiple confounding variables. Confounding variables were selected by clinical experience and stepwise regression (*p* < 0.05 for selection), and variables with variance inflation factors greater than 5 were excluded from the model. Kaplan-Meier survival analysis was used to assess 28-day mortality, and incidence of AKI based on GNRI strata, and differences between groups were assessed by Log-Rank test. The dose-response relationship between GNRI and 28-day mortality was investigated using restricted cubic splines (RCS). Subgroup analysis and interaction analysis were performed according to age, gender, BMI, heart failure, CKD, sepsis, and hypertension. In addition, multivariate linear regression was used to analyze the relationship between GNRI and length of hospital stay.

R software (Version 4.2.0) and STATA software (Version 16.0) were used for data analysis and visualization. A two-tailed test indicated that *P* < 0.05 was considered statistically significant.

## 3 Results

### 3.1 Baseline characteristics

**Figure 1:**
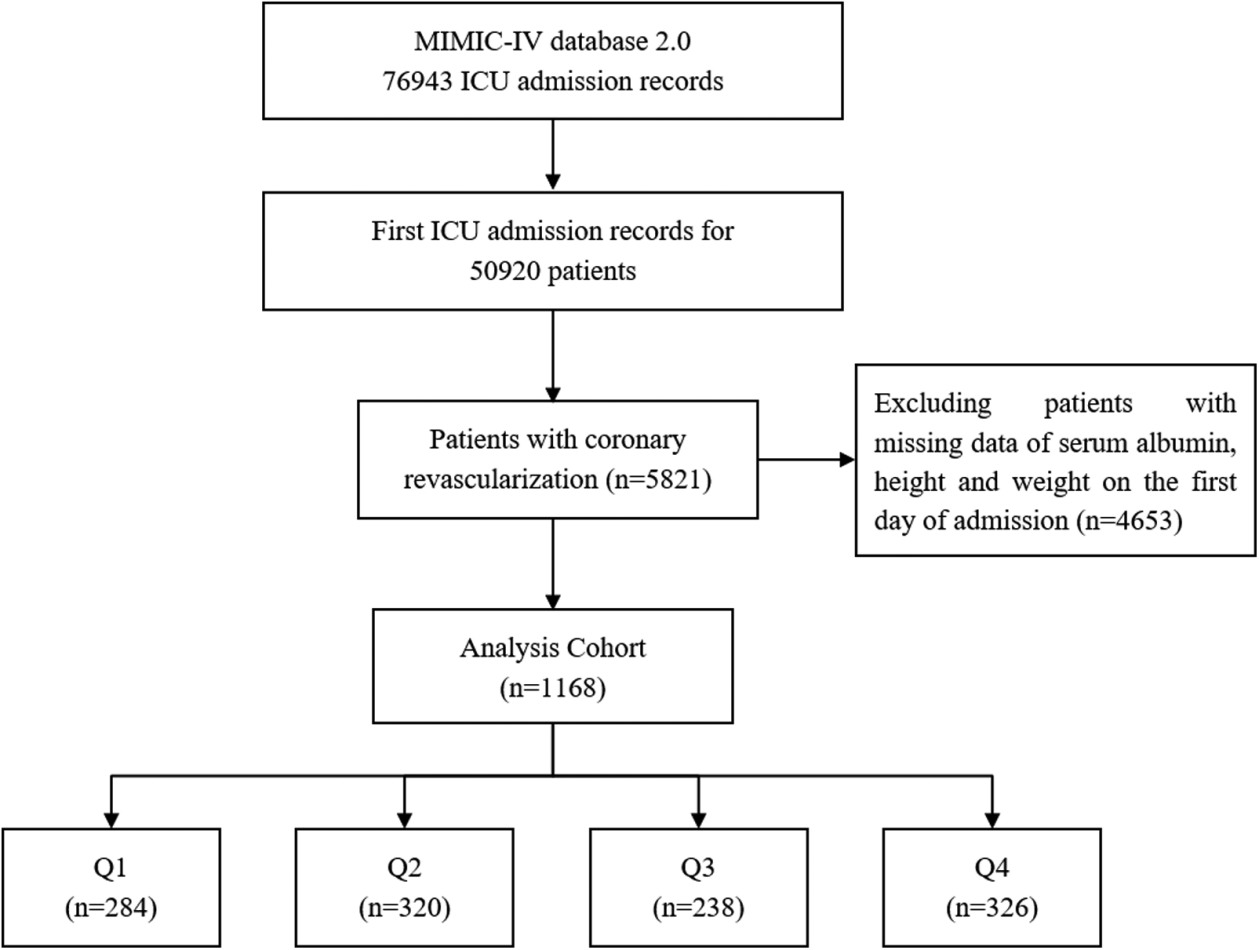
Screening flow chart.

A total of 1,168 patients with coronary revascularization were enrolled in this study. The screening flow chart is shown in Figure 1.

The baseline characteristics of the four groups classified according to GNRI quartiles are shown in Table 1. Those with lower GNRI tended to be older, more female, had higher SOFA scores, higher prevalence of heart failure, CKD, sepsis, and use of RRT, diuretics, and vasoactive drugs. In laboratory indexes, RBC, hemoglobin, platelet, serum albumin, and sodium in patients with lower GNRI were lower than those in patients with higher GNRI, while WBC, BUN, and potassium were on the contrary. In addition, those with lower GNRI had higher 28-day mortality, greater risk of AKI, and longer hospital stays.

**Table 1:** Baseline characteristics according to GNRI quartiles.

Variables	Overall *n* = 1,168	*Q*1 (<86.37) *n* = 284	*Q*2 (86.37–90.84) *n* = 320	*Q*3 (90.85–96.78) *n* = 238	*Q*4 (≥96.79) *n* = 326	*P*-value
Age (years)	70.7 (62.0, 78.5)	72.3 (63.5, 79.6)	71.1 (63.4, 79.3)	70.7 (62.5, 78.1)	68.3 (59.5, 75.5)	<0.001
Male, *n* (%)	817 (70.0)	166 (58.5)	229 (71.6)	172 (72.3)	250 (76.7)	<0.001
BMI (kg/m^2^)	28.4 (24.9, 32.3)	28.2 (23.6, 32.2)	28.6 (25.3, 32.4)	28.5 (25.8, 32.3)	28.1 (25.0, 32.4)	0.164
SOFA	5.0 (3.0, 8.0)	7.0 (4.0, 10.0)	6.0 (3.5, 9.0)	5.0 (2.0, 7.0)	4.0 (2.0, 7.0)	<0.001
Comorbidities, *n* (%)						
Heart failure	486 (41.6)	147 (51.8)	138 (43.1)	91 (38.2)	110 (33.7)	<0.001
AMI	413 (35.4)	96 (33.8)	88 (27.5)	88 (37.0)	141 (43.3)	<0.001
CKD	285 (24.4)	90 (31.7)	91 (28.4)	46 (19.3)	58 (17.8)	<0.001
Hypertension	572 (49.0)	130 (45.8)	135 (42.2)	134 (56.3)	173 (53.1)	0.002
DM	254 (21.8)	68 (23.9)	74 (23.1)	57 (24.0)	55 (16.9)	0.094
Sepsis	665 (56.9)	191 (67.3)	201 (62.8)	115 (48.3)	158 (48.5)	<0.001
Malignancy	192 (16.4)	47 (16.6)	53 (16.6)	41 (17.2)	51 (15.6)	0.967
Interventions, *n* (%)						
RRT	114 (9.8)	59 (20.8)	28 (8.8)	11 (4.6)	16 (4.9)	<0.001
Diuretics	855 (73.2)	225 (79.2)	259 (80.9)	179 (75.2)	192 (58.9)	<0.001
Vasoactive drugs	452 (38.7)	144 (50.7)	132 (41.3)	76 (31.9)	100 (30.7)	<0.001
PCI	353 (30.2)	67 (23.6)	69 (21.6)	76 (31.9)	141 (42.3)	<0.001
CABG	821 (70.3)	220 (77.5)	253 (79.1)	162 (68.1)	186 (57.1)	<0.001
Laboratory tests						
RBC (m/ul)	3.4 (2.8, 4.0)	3.0 (2.5, 3.5)	3.2 (2.7, 3.6)	3.4 (3.0, 3.9)	4.1 (3.5, 4.6)	<0.001
WBC (K/ul)	12.2 (9.1, 16.4)	13.0 (10.0, 17.0)	13.0 (9.8, 17.6)	12.7 (9.2, 16.7)	10.7 (8.0, 14.4)	<0.001
Hemoglobin (g/dl)	10.7 (8.8, 12.8)	9.3 (7.7, 10.9)	10.0 (8.6, 12.0)	11.0 (9.2, 12.8)	12.7 (10.5, 14.0)	<0.001
Platelet (K/ul)	171.0 (131.0, 221.0)	148.0 (111.0,197.0)	154.5 (125.0, 204.5)	176.0 (138.0, 222.0)	198.5 (158.0, 256.0)	<0.001
Scr (mg/dl)	1.0 (0.8, 1.3)	1.0 (0.8, 1.5)	1.0 (0.8, 1.3)	1.0 (0.8, 1.2)	1.0 (0.8, 1.2)	0.087
BUN (mg/dl)	18.0 (14.0, 25.0)	20.0 (15.0, 29.0)	18.0 (14.0, 25.0)	17.0 (14.0, 22.0)	17.5 (13.0, 23.0)	<0.001
Albumin (g/dl)	3.3 (3.0, 3.7)	2.7 (2.5, 2.9)	3.2 (3.1, 3.3)	3.5 (3.4, 3.6)	3.9 (3.8, 4.2)	<0.001
Glucose (mg/dl)	138.0 (115.5, 173.5)	143.0 (120.0, 184.5)	138.0 (113.0, 169.0)	133.0 (115.0, 169.0)	137.0 (112.0, 169.0)	0.070
Potassium (mmol/L)	4.4 (3.9, 4.9)	4.6 (4.0, 5.2)	4.5 (4.1, 5.0)	4.3 (3.9, 4.9)	4.2 (3.8, 4.7)	<0.001
Sodium (mmol/L)	136.0 (134.0, 138.0)	135.0 (133.0, 138.0)	136.0 (134.0, 138.0)	136.5 (134.0, 138.0)	137.0 (135.0, 139.0)	<0.001
Events						
28-day mortality, *n* (%)	101 (8.7)	44 (15.5)	25 (7.2)	14 (5.9)	20 (6.1)	<0.001
AKI, *n* (%)	561 (48.1)	177 (62.3)	163 (50.9)	90 (37.8)	131 (40.2)	<0.001
Hospital stays (days)	8.2 (5.4, 13.1)	10.8 (7.2, 18.1)	9.2 (6.2, 12.9)	7.8 (5.3, 12.5)	6.0 (4.1, 10.1)	<0.001

SOFA, sequential organ failure assessment; CKD, chronic kidney disease; DM, diabetes mellitus; RRT, renal replacement therapy; RBC, red blood cell; WBC, white blood cell; SCr, serum creatinine; BUN, blood urea nitrogen; AKI, acute kidney injury. AMI: Acute Myocardial Infarction; PCI: Percutaneous Coronary Intervention; CABG: Coronary Artery Bypass Grafting.

### 3.2 Primary endpoint

**Figure 2:**
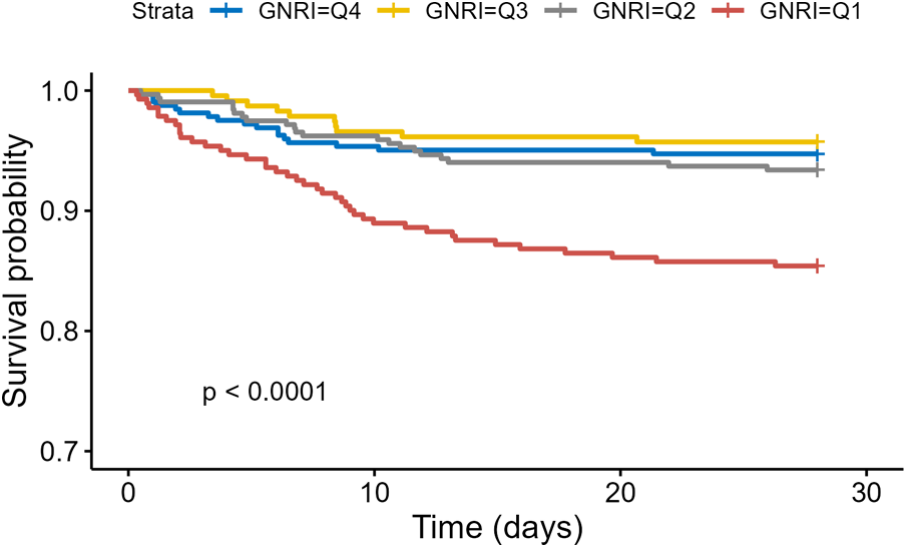
Kaplan–Meier survival analysis of 28-Day mortality based on GNRI stratification.

Of the 1,168 patients, 101 died within 28 days, accounting for 8.7%. Kaplan–Meier curves (Figure 2) showed a significant increase in 28-day mortality for GNRI <86.37 (Log-rank *P* <0.0001).

As shown in Table 2, both univariate and multivariate Cox regression models confirmed the independent effect of GNRI on 28-day mortality in patients with coronary revascularization. When GNRI is treated as a continuous variable, there is no significant difference between the unadjusted model and the unadjusted model. [HR, 0.93 (95% CI: 0.91, 0.96), *P* < 0.001], partially adjusted model GNRI was significantly negatively associated with 28-day mortality in both the fully adjusted model [HR, 0.94 (95% CI: 0.92, 0.96), *P* < 0.001] and the fully adjusted model [HR, 0.95 (95% CI: 0.93, 0.97), *P* < 0.001]. When GNRI was a categorical variable, the low GNRI group (*Q*1) was significantly associated with an increased risk of 28-day mortality: Unadjusted model [HR, 2.64 (95% CI: 1.56, 4.48), *P* < 0.001], partially adjusted model [HR, 2.30 (95% CI: 1.35, 3.92), *P* = 0.002], and fully adjusted model [HR, 1.97 (95% CI: 1.12, 3.46), *P* = 0.019].

**Table 2:** Cox proportional hazard ratios for 28-day mortality.

Categories	Model 1 HR (95% CI)	*P-*value	Model 2 HR (95% CI)	*P-*value	Model 3 HR (95% CI)	*P-*value
GNRI	0.93 (0.91, 0.96)	<0.001	0.94 (0.92, 0.96)	<0.001	0.96 (0.93, 0.98)	<0.001
GNRI (category)						
*Q*1 (<86.37)	2.64 (1.56, 4.48)	<0.001	2.30 (1.35, 3.92)	0.002	1.82 (1.04, 3.21)	0.037
*Q*2 (86.37–90.84)	1.16 (0.64, 2.12)	0.618	1.07 (0.59, 1.95)	0.821	0.96 (0.52, 1.76)	0.883
*Q*3 (90.85–96.78)	0.94 (0.48, 1.87)	0.868	0.88 (0.45, 1.75)	0.724	1.01 (0.51, 2.00)	0.984
*Q*4 (≥96.79)	Ref.		Ref.		Ref.	
*P* for trend		<0.001		0.001		0.035

Model 1 was unadjusted.

Model 2 was adjusted for sex, age, and BMI.

Model 3 was adjusted for sex, age, BMI, Scr, hemoglobin, vasoactive drugs, diuretics, hypertension, heart failure, DM, CKD, AMI, sepsis, and malignancy.

**Figure 3:**
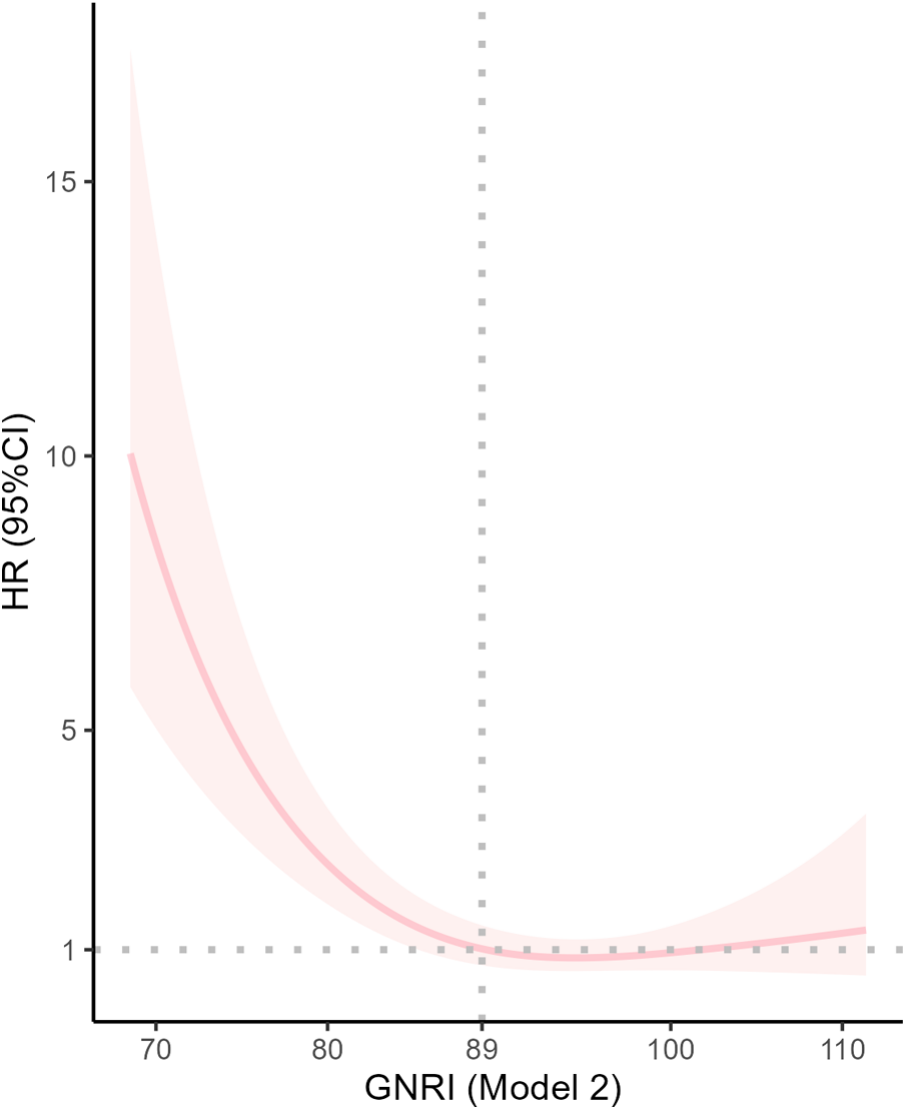
Dose response curves of GNRI and 28-Day mortality in patients undergoing coronary revascularization.

RCS curves (Figure 3) showed a nonlinear association between GNRI and 28-day mortality after adjusting for some confounders (*P* for non-linearity <0.001).

**Figure 4:**
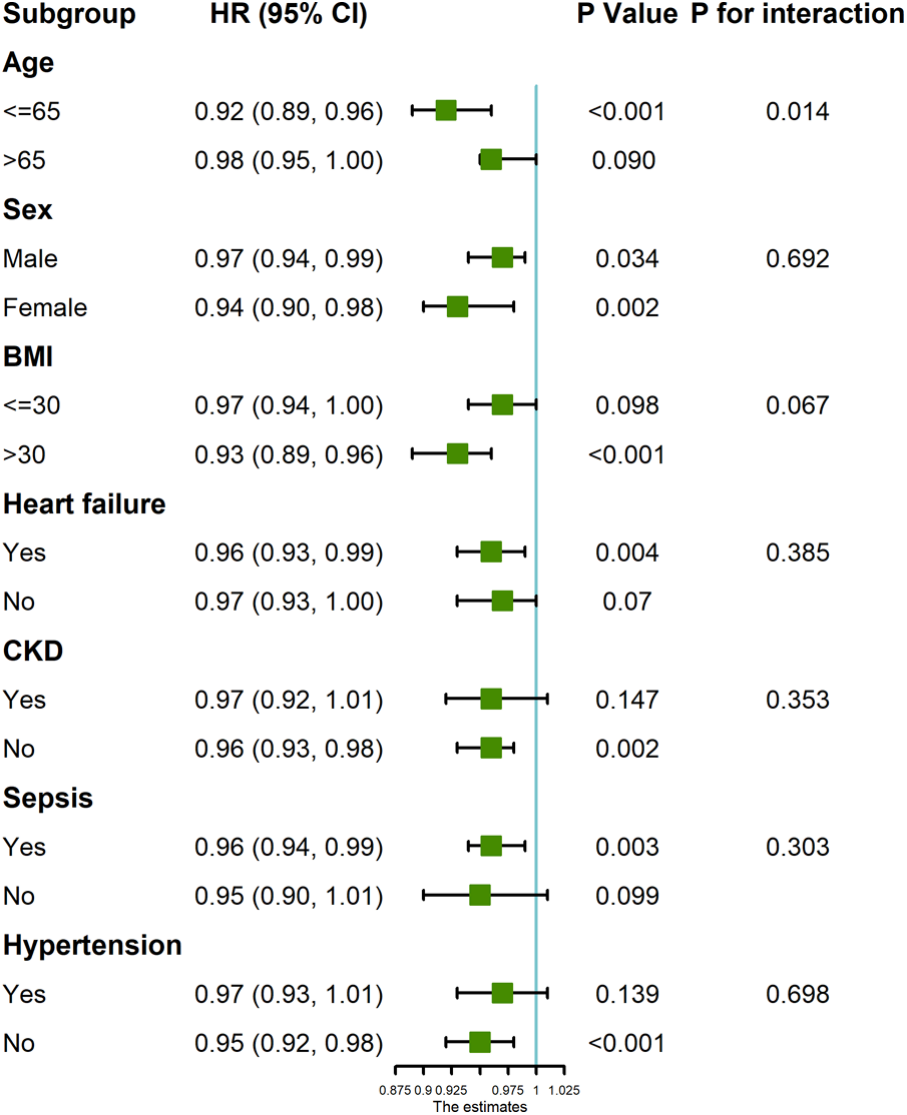
Forest plot of GNRI score and 28-day mortality in patients undergoing coronary revascularization.

Subgroup analysis (Figure 4) showed a robust independent association between GNRI and 28-day mortality. Of note, GNRI had interaction effects with age (*P* for interaction = 0.016) and BMI (*P* for interaction = 0.024).

### 3.3 Secondary endpoints

**Figure 5:**
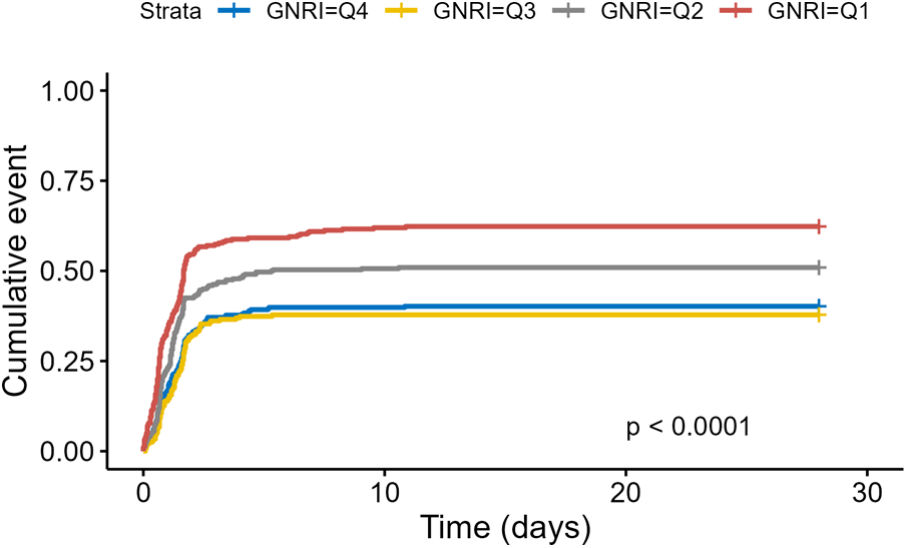
Kaplan–Meier survival analysis of incidence of AKI based on GNRI stratification.

We further assessed the effect of GNRI on the incidence of AKI and length of stay in the study population. Figure 5 showed that cumulative AKI incidence was significantly higher for lower GNRI (*Q*1, *Q*2) (Log-rank *P* < 0.0001). Cox regression models (Table 3) also revealed a significant negative correlation between GNRI and AKI incidence when GNRI was a continuous variable (*P* < 0.001).

**Table 3:** Cox proportional hazard ratios for AKI incidence.

Categories	Model 1 HR (95% CI)	*P-*value	Model 2 HR (95% CI)	*P-*value	Model 3 HR (95% CI)	*P-*value
GNRI	0.96 (0.95, 0.97)	<0.001	0.96 (0.95, 0.97)	<0.001	0.97 (0.96, 0.99)	<0.001
GNRI (category)						
*Q*1 (<86.37)	1.93 (1.54, 2.43)	<0.001	1.86 (1.47, 2.34)	<0.001	1.42 (1.11, 1.82)	0.005
*Q*2 (86.37–90.84)	1.38 (1.10, 1.74)	0.006	1.30 (1.03, 1.64)	0.026	1.05 (0.83, 1.34)	0.668
*Q*3 (90.85–96.78)	0.92 (0.71, 1.21)	0.569	0.88 (0.67, 1.15)	0.359	0.86 (0.65, 1.13)	0.277
*Q*4 (≥96.79)	Ref.		Ref.		Ref.	
*P* for trend		<0.001		<0.001		0.002

Model 1 was unadjusted.

Model 2 was adjusted for sex, age, and BMI.

Model 3 was adjusted for sex, age, BMI, hemoglobin, vasoactive drugs, diuretics, hypertension, heart failure, DM, CKD, AMI, sepsis, and malignancy.

In addition, when GNRI was categorically stratified, the low GNRI group (*Q*1) was significantly associated with the risk of AKI: Unadjusted model [HR, 1.93 (95% CI: 1.54, 2.43), *P* < 0.001], partially adjusted model [HR, 1.86 (95% CI: 1.47, 2.34), *P* < 0.001], and fully adjusted model [HR, 1.40 (95% CI: 1.10, 1.79), *P* = 0.007].

Multiple linear regression models (Table 4) showed that the lower the GNRI, the longer the length of hospital stay, both as continuous and categorical variables.

**Table 4:** Multiple linear regression of the association between GNRI and length of stay.

Categories	Model 1 *β* (95% CI)	*P-*value	Model 2 *β* (95% CI)	*P-*value	Model 3 *β* (95% CI)	*P-*value
GNRI	−0.23 (−0.30, −0.17)	<0.001	−0.23 (−0.29. −0.16)	<0.001	−0.07 (−0.14, −0.01)	0.003
GNRI (category)						
*Q*1 (<86.37)	5.80 (4.32, 7.28)	<0.001	5.64 (4.15, 7.14)	<0.001	2.16 (0.66, 3.66)	0.005
*Q*2 (86.37–90.84)	2.21 (0.78, 3.64)	0.002	2.03 (0.59, 3.47)	0.006	−0.46 (−1.87, 0.93)	0.511
*Q*3 (90.85–96.78)	1.15 (−0.40, 2.70)	0.145	1.00 (−0.55, 2.55)	0.207	0.04 (−1.42, 1.49)	0.960
*Q*4 (≥96.79)	Ref.		Ref.		Ref.	
*P* for trend		<0.001		<0.001		0.021

Model 1 was unadjusted.

Model 2 was adjusted for sex, age, and BMI.

Model 3 was adjusted for sex, age, BMI, Scr, hemoglobin, vasoactive drugs, diuretics, hypertension, heart failure, DM, CKD, AMI, sepsis, and malignancy.

## 4 Discussion

Coronary revascularization is a cornerstone in the treatment of coronary heart disease; however, this procedure is not without its risks, as it is associated with a certain mortality rate and potential complications. Consequently, there is an imperative need to identify risk factors that influence postoperative prognosis. Malnutrition has been recognized as a significant predictor of adverse outcomes in various diseases, and GNRI is a widely utilized tool for assessing nutritional status. This study investigated the association between GNRI and key clinical outcomes, including mortality, the incidence of AKI, and the duration of hospitalization, in patients undergoing coronary revascularization. The findings revealed that patients with low GNRI scores exhibited higher mortality rates, an increased incidence of AKI, and prolonged hospital stays compared to those with high GNRI scores.

Malnutrition is a predictor of multiple diseases and surgical outcomes and is estimated to affect approximately 30% to 70% of hospitalized patients (9). The study found that approximately 20% of patients undergoing cardiac surgery experience weight loss during the postoperative period, and stress reactions to surgery and other comorbidities are potential factors that cause weight loss and lead to a malnourished state (10). Several nutritional indices have been developed to assess nutritional status, among which GNRI, which takes into account both serum albumin levels and body weight, is accurate comprehensive, and widely used. This study confirms the independent effect of GNRI on 28-day mortality in patients with coronary revascularization. This is consistent with the results of current studies. For example, Kanda et al. (11) evaluated the correlation between all-cause death and malnutrition in 268 AMI patients undergoing PCI and found that GNRI can predict the mortality rate within 1 month after PCI in AMI patients. Malnutrition may be associated with short-and long-term poor prognosis in AMI patients after PCI. Noike et al. (12) found that frailty and malnutrition were independent predictors of MACE in patients undergoing PCI for stable angina and were detrimental to prognosis. Similar results were found in CABG patients. Tasbulak et al. (13) found a strong correlation between nutritional status and long-term major adverse cardiovascular events (MACCE) and mortality in CABG patients, with CABG patients with low GNRI having higher mortality. In addition, an observational study showed that patients undergoing coronary artery bypass grafting with reduced GNRI had increased rates of MACE and decreased survival during long-term follow-up (14).

In addition to the association between GNRI and mortality, the study also found a significantly higher incidence of AKI in patients with lower GNRI and Cox regression models revealed a significant negative correlation between GNRI and AKI incidence. AKI is one of the possible complications of coronary revascularization surgery (15). The etiology is complex and unknown. Concomitant AKI increases the incidence of adverse medical events in patients undergoing surgery. Acute kidney injury (AKI) after PCI has been reported to be associated with significantly increased short-and long-term mortality and loss of renal function (16). One study showed that patients undergoing CABG had a higher risk of AKI than PCI intervention, but regardless of the revascularization strategy used, the impact of concomitant AKI on prognosis was significant (17). Aykut et al. (18) found that malnutrition and weakness were closely associated with AKI after CABG.

Finally, multiple linear regression models also showed that the lower the GNRI, the longer the length of hospital stay. Malnutrition affects the prognosis. Well-nourished patients recover from disease faster, resulting in faster discharge and reduced hospital costs.

## 5 Limitations

Inevitably, this study has its limitations: Firstly, the study employed an observational design, which does not allow for complete control over potential confounding variables, and this may affect the interpretation of treatment effects. Secondly, the data source is from a single medical center, which may limit the generalizability and external applicability of the results. Additionally, the study only included critically ill patients undergoing revascularization in the ICU, failing to cover a broader patient population, which may affect the comprehensive understanding of treatment effects. Lastly, due to the lack of randomization, it is impossible to fully determine whether the observed treatment effects are indeed caused by the treatment itself or other unmeasured factors. To overcome these limitations, future studies can adopt a multi-center design, randomized controlled trials, and include a wider range of patient populations to more accurately assess treatment effects.

## 6 Conclusion

Nutritional level is associated with prognosis in patients undergoing coronary revascularization, and GNRI can be used as a predictor of postoperative mortality. This study shows that patients with low GNRI have higher mortality, AKI incidence, and longer hospital stays, suggesting that we can effectively predict prognosis after coronary revascularization by evaluating GNRI score, to implement relevant interventions to reduce postoperative mortality.

## Data Availability

The original contributions presented in the study are included in the article/Supplementary Material, further inquiries can be directed to the corresponding author.
